# Eco‐Evolutionary Dynamics of Anadromous Alewife Restoration in a Whole‐Ecosystem Experiment

**DOI:** 10.1111/eva.70350

**Published:** 2026-11-06

**Authors:** Kerry Reid, Katherine A. Littrell, Andrew D. MacDonald, Kevin Job, Stephen R. Gephard, John Carlos Garza, David M. Post, Eric P. Palkovacs

**Affiliations:** ^1^ Institute of Marine Sciences University of California Santa Cruz California USA; ^2^ Department of Ecology and Evolutionary Biology Yale University New Haven Connecticut USA; ^3^ Fisheries Division Connecticut Department of Energy and Environmental Protection Old Lyme Connecticut USA; ^4^ Southwest Fisheries Science Center National Marine Fisheries Service Santa Cruz California USA; ^5^ Department of Ocean Sciences University of California Santa Cruz California USA; ^6^ Department of Ecology and Evolutionary Biology University of California Santa Cruz California USA

**Keywords:** connectivity, hybridization, intraspecific variation, management experiment, resident ecotype, river herring

## Abstract

Restoration efforts can serve as opportunities to conduct ecological and evolutionary experiments at the whole‐ecosystem scale. We evaluated the eco‐evolutionary dynamics of restoring anadromous alewives ( *Alosa pseudoharengus* ) to a lake occupied by a well‐established landlocked population isolated ca. 350 years ago. The restoration of Rogers Lake involved the construction of fishways on all downstream dams and the annual stocking of anadromous adults. Throughout the restoration project, we quantified juvenile recruitment, abundance, and growth, as well as hybridization and introgression with the landlocked population. Restoration led to the annual production of anadromous juveniles and the natural return of anadromous adults. During the decade of restoration, > 27,000 anadromous adults ascended the fishway, with ~78% identified as offspring of stocked parents. Juvenile anadromous abundance fluctuated annually but never exceeded 8% of the total juvenile composition. Anadromous juveniles in Rogers Lake grew faster than those in nearby Bride Lake, the source of the stocked anadromous adults, and matched landlocked and hybrid growth rates. Parentage analyses revealed that stocking date influenced reproductive success, age‐of‐return, and day‐of return. Hybrid juveniles appeared early in the restoration and increased in abundance. Hybrids exhibited both life histories, with the majority remaining in the lake as freshwater residents, but several returning as mature anadromous adults. Landlocked juvenile abundance declined, suggesting ongoing demographic shifts through the restoration. Overall, restoration efforts reestablished the anadromous life history in the lake but also led to hybridization between forms. These results provide an example of how evolutionary processes can shape the outcome of restoration efforts.

## 1 Introduction

Restoration efforts provide important opportunities to conduct ecological and evolutionary experiments at the whole‐ecosystem scale (Young et al. 2005; LaRue et al. 2017; Wainwright et al. 2018; Hendry et al. 2024). The goal of ecological restoration is often to rebuild habitat quality and connectivity so that species can establish and persist as self‐sustaining populations. Restoration efforts may commonly shape evolutionary processes such as genetic drift, selection, and gene flow, which may in turn influence ecological processes such as population growth rate and species interactions (Stockwell et al. 2016). Thus, evolution may be an important determinant of restoration success (or failure). This may be especially true in the context of secondary contact, which occurs when connectivity is reestablished between divergent lineages and can lead to either introgression or reinforcement (Coyne and Orr 2004; Skúlason et al. 2019).

Anadromous fish populations in North America have shown substantial declines in the last century and are < 10% of their former population sizes (Waldman and Quinn 2022). Various ecological restoration activities have been implemented to allow anadromous species access to suitable spawning and rearing habitats, with a major effort focused on removing or circumventing dams impeding access (Pess et al. 2014; Reid et al. 2020; Waldman and Quinn 2022; Zarri et al. 2022). Dam removal and fishway construction to restore anadromous fish populations are common restoration approaches that may shape the ecological and evolutionary dynamics of secondary contact. The secondary contact created by these management actions can lead to, hybridization and introgression (Hitt et al. 2003; Muhlfeld et al. 2009; Reid et al. 2020), altered life histories (Bolstad et al. 2017; Heino et al. 2021), and reduced fitness (Muhlfeld et al. 2009), all of which can impact restoration success.

Here we used the restoration of Rogers Lake (Old Lyme, Connecticut, USA) as an opportunity to study the eco‐evolutionary dynamics of restoring an anadromous alewife ( *Alosa pseudoharengus* ) run into a lake where a landlocked population of alewife was already established (Palkovacs et al. 2008; Post et al. 2008; Littrell et al. 2018; Reid et al. 2020). Alewives exhibit complex life histories that have been significantly shaped by both natural behaviors and human‐induced environmental change. There are two ecotypes of alewife: the ancestral migratory (anadromous) form and a landlocked (freshwater resident) form. Anadromous alewives inhabit the Atlantic coast of North America from Canada to South Carolina (Atlantic States Marine Fisheries Commission (ASMFC) 2012). The anadromous form migrates in spring to freshwater to spawn and the juveniles rear during summer in freshwater lakes before migrating to the ocean. Anadromous alewives typically spawn as 3‐ to 5‐year‐olds as males and 4‐ to 6‐year‐olds as females (Atlantic States Marine Fisheries Commission (ASMFC) 2012), with iteroparity rates in natural populations thought to be as high as 30% (Davis and Schultz 2009; Spares et al. 2023). However, recent declines in maturation age and repeat spawning have been observed (Palkovacs, Hasselman, et al. 2014). Historically, anadromous alewives supported targeted fisheries, but their populations have experienced significant and persistent declines since the 1970s. These anadromous populations exhibit regional genetic structure (Palkovacs, Mandeville, and Post 2014; McBride et al. 2015; Baetscher et al. 2017; Reid et al. 2018), localized straying (Reid et al. 2018), and evidence of local adaptation (McBride et al. 2015). Conservation efforts have increasingly focused on reducing bycatch, as river herring are frequently caught in mackerel and Atlantic herring fisheries, particularly from rivers within southern New England (Hasselman et al. 2016; Reid et al. 2023). Additionally, the expansion of fish‐passage projects in southern New England is facilitating the restoration of migratory routes for anadromous species and is leading to the possibility of secondary contact between anadromous and landlocked forms (Littrell et al. 2018; Reid et al. 2020).

While many inland landlocked populations were established through translocations after the mid‐1800s, Palkovacs et al. (2008) identified distinct coastal populations that were independently isolated and found above colonial‐era dams. Following these landlocking events, alewives have evolved physiological, ecological, and foraging traits that differ significantly from those of their anadromous progenitors (Palkovacs et al. 2008; Palkovacs, Hasselman, et al. 2014; Velotta et al. 2017). The landlocked alewife form spends its entire life cycle in freshwater and spawns during spring, with juveniles rearing in summer. Spawning phenology varies between anadromous and landlocked forms, and overlap of spawning duration has been estimated between 3% and 13% depending on the lake pair and year compared (Littrell et al. 2018). In freshwater systems, their altered foraging behavior is driven by shifts in prey availability, reshaping zooplankton communities (Post et al. 2008; Palkovacs and Post 2009), and feeds back to further reinforce divergence in foraging traits (Palkovacs and Post 2008; Post and Palkovacs 2009; Palkovacs, Mandeville, and Post 2014). The divergence in life‐history traits, physiology, behavior, and ecological impacts between forms provides an interesting setting to study secondary contact through restoration. Reid et al. (2020) observed that within just the first three years of secondary contact between anadromous and landlocked alewife ecotypes, anadromous juvenile production was detected, as well as the first discrete hybrid classes. However, questions remain about the longer‐term eco‐evolutionary consequences of secondary contact and the overall restoration of the anadromous life history form.

Here we use the restoration of anadromous alewife to Rogers Lake as a whole‐ecosystem experiment to ask: (1) how does anadromous alewife restoration through stocking influence successful recruitment and contribute to a self‐sustaining population, and (2) how does the restoration of anadromous alewives interact with the resident landlocked population?

## 2 Materials and Methods

### 2.1 Reestablishing the Anadromous Alewife Run

There are three dams on Mill Brook between Rogers Lake (41.3582° N, 72.2977° W) and the Connecticut River (Figure 1). The effort to restore connectivity to Rogers Lake was completed in 2014 following the installation of the final fishway at the Rogers Lake dam. To reestablish a self‐sustaining anadromous run into Rogers Lake (Figure 1A), prespawn adult alewives were released annually, 2015–2023, to promote a run with overlapping generations (Figure 1B). In 2015, 134 adult alewives returning from the ocean were captured after passing the lowest most fishway on Mill Brook (41.3379° N, 72.3209° W) and stocked into Rogers Lake. Due to the very small run size in Mill Brook (Figure S1), from 2016 to 2023, fish were captured from Bride Lake (41.3276° N, 72.2378° W) and stocked into Rogers Lake (Figure 2A; 24,832 fish). Bride Lake was chosen as the source of stocked fish due to the geographic proximity of the lake (Figure 1A), a substantially larger annual run size (Figure S1), and the genetic similarity between Mill Brook and Bride Lake fish (Reid et al. 2018). Both Bride Lake and the Connecticut River flow into Long Island Sound, an estuary of the Atlantic Ocean. In 2015 through 2018, stocked fish were sexed, length was measured, and a fin clip was taken for genetic analyses (Tables 1 and 2). Fin tissue was placed on blotting paper to dry and then stored in coin envelopes. Adult fish stocked in 2015–2017 had previously been genotyped with a microhaplotype panel suitable for categorical genetic assignments and high‐resolution parentage analysis (Reid et al. 2020).

**FIGURE 1:**
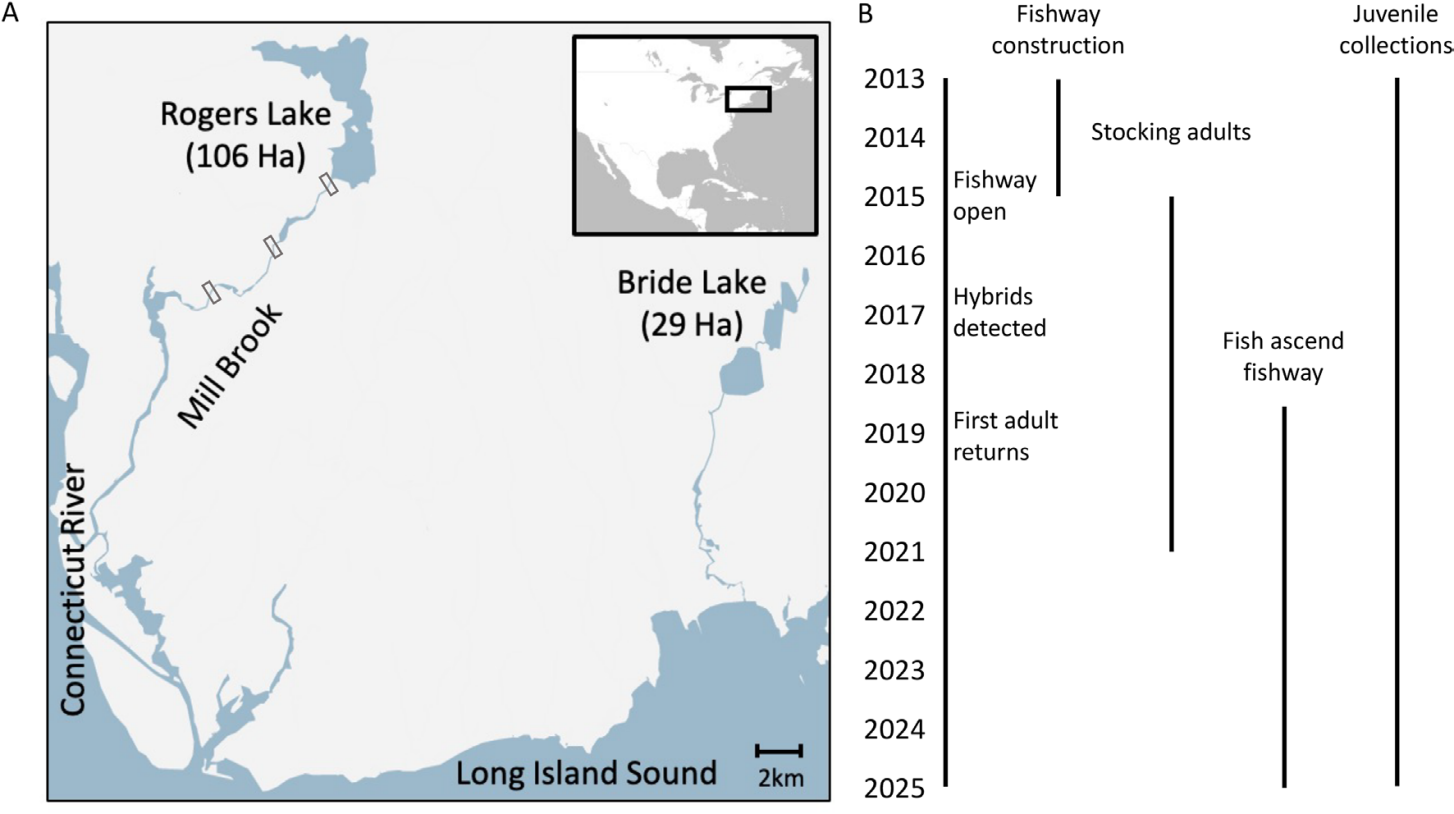
(A) Map of Old Lyme Connecticut, with Rogers and Bride Lake highlighted, inset is North America, indicating the region of the study area. During 9 years (2015–2023), 24,832 adult fish were stocked in Rogers Lake from Mill Brook and Bride Lake to reestablish the anadromous run to Rogers Lake. Rogers Lake connects to the Connecticut River via Mill Brook and is part of Long Island Sound, which connects to the Atlantic Ocean. Blocks along Mill Brook indicate the position of the three fishways, the second fishway is at Mary Steube where the fish were translocated from in 2015. (B) Timeline of experiment of major events and duration of stocking and sampling.

**FIGURE 2:**
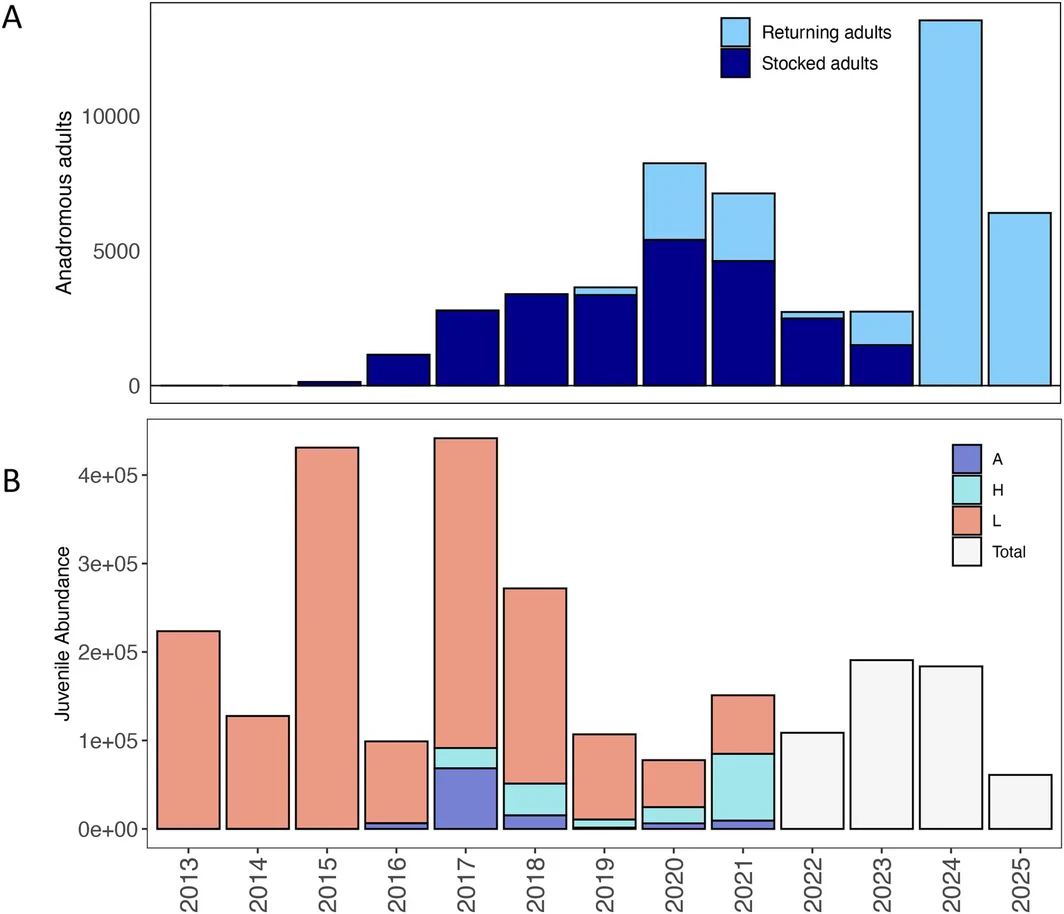
Anadromous adults entering Rogers Lake and annual juvenile production. (A) Number of stocked anadromous fish between 2015 and 2023 and naturally returning adults between 2019 to 2025. (B) Estimated juvenile abundance in Rogers Lake each August for anadromous (A), hybrids (H) and landlocked juveniles (L). For years where we do not have genetic ancestry data to estimate the abundance composition, we report the abundance totals.

**TABLE 1:** Sampling numbers of stocked adults, juveniles, and migrating adults collected at the Rogers Lake fishway (2019–2022) for which genetic analyses were conducted through the duration of the study period.

Year	Stocked adults	Sampled juveniles	Migrating adults
2013		120	
2014		65	
2015	134	438	
2016	1144	1115	
2017	2787	1409	
2018	3392	1635	
2019		753	286
2020		923	568
2021		1154	501
2022			13
Total	7457	7612	1368

**TABLE 2:** Reproductive output by year and stocking date.

Year	Source	Stocking date	F	M	Total	Yearly total	F‐rep	M‐rep	Parents	Offs
2015	Mary Steube	15‐Apr‐15	9	27	36		1	1		
		17‐Apr‐15	1	2	3					
		1‐May‐15	26	63	89					
		13‐May‐15	1	5	6	134			2	1
2016	Bride Lake	2‐May‐16	369	207	576		2	2		
		4‐May‐16	344	224	568	1144	2	2	8	4
2017	Bride Lake	30‐Mar‐17	428	576	1004		172	240		
		12‐Apr‐17	399	492	891		209	202		
		19‐Apr‐17	339	553	892	2787	66	72	961	1115
2018	Bride Lake	3‐Apr‐18	609	723	1336 (4 NS)		13	18		
		12‐Apr‐18	449	626	1075		24	36		
		20‐Apr‐18	470	511	981	3392	28	19	138	80
Total			3444	4009	6121	7457	517	592	1109	1200

Abbreviations: F, female; F‐rep, number of females contributing to offspring; M, male; M‐rep, number of males contributing to offspring; NS, no sex known; Offs, offspring.

### 2.2 Juvenile Sampling in Rogers Lake

To determine whether the stocking of anadromous alewife led to spawning and the production of juveniles, we collected juveniles each August. The August sampling represents fish that have survived to the end of summer and coincides with the main period of outmigration for anadromous juveniles. We started collecting juveniles in 2013 (two years before stocking began) and extended the sampling through the initiation of secondary contact (2015–2025) (Table S1). Whole specimens were collected, and we measured length and weight of each individual fish and took a fin clip for genetic analyses. Genomic DNA was extracted using the DNeasy Blood and Tissue Kit (Qiagen) from all juveniles collected from 2013 to 2021 (7660 in total). Amplicons targeting 114 loci were amplified and sequenced with 2 × 75 bp protocol on a MiSeq platform (Illumina Inc.) (Reid et al. 2020). The R package “*microhaplot*” (https://doi.org/10.5281/zenodo.820110) R v. 3.4.4 (R Core Development Team 2018) was used to call haplotypes for each locus and then haplotypes were turned into genotypes. Individuals were discarded if they had more than 20% missing data across all loci, with 7612 samples retained for further analyses (Table 1).

We used NEWHYBRIDS v. 1.0 (Anderson and Thompson 2002) to assign juveniles as pure landlocked, pure anadromous, or to one of four hybrid classes (F1, F2, anadromous and landlocked backcrosses) (burn‐in of 10,000 replicates and 20,000 additional iterations). The ancestry of juveniles was further confirmed using STRUCTURE v. 2.3.4 (Pritchard et al. 2000), with *K* = 2, and a 50,000 burn‐in and 150,000 retained iterations. The 2018 stocked anadromous adults that had not previously been sequenced, were also sequenced and processed as described above. We then identified the stocked fish (2015–2018) that produced the putatively identified F1 and anadromous juveniles in the lake with a maximum‐likelihood parentage analysis using the software FRANz (v. 2.0.0; Riester et al. 2009). The settings included analyses run with and without prior information, with the prior information including the stocking year of adults and phenotypic sex. Parent‐offspring assignments were retained if the assignments had a posterior probability > 0.98 and LOD score > 10.

### 2.3 Annual Juvenile Abundance

To estimate the number of anadromous juveniles being produced by stocked adults in Rogers Lake annually, we first estimated the overall abundance of juveniles in the lake, from 2013, before the restoration started, to 2025. In brief, to derive abundance estimates of all juveniles in the lake, density was calculated based on replicate sampling sets which were taken at night using a purse seine that encircled an area of ~100m^2^, which was previously described for Rogers Lake (Littrell et al. 2018; Reid et al. 2020). In each case, three replicates were taken in littoral and pelagic habitats in the lake and estimates were extrapolated based on the area of each habitat (106 Ha, with ~50% littoral and 50% pelagic habitat) from 2013 to 2017 (Reid et al. 2020). From 2018, we increased the replicates to six in each habitat. We then specifically estimated the abundance of the anadromous ecotype from these estimates each summer based on the estimated abundance estimates and the proportions of anadromous juveniles genetically identified in years when they were detected (2016–2021). We compared these estimates to abundance estimates collected in the same manner from Bride Lake from 2013 to 2024.

We also estimated the abundance of hybrid and landlocked juveniles in the lake. For hybrids, this was from the secondary contact and for landlocked juveniles, density and abundance estimates were available from 2013 to 2021. To quantify the demographic trajectory of the landlocked population, we assessed the difference in landlocked abundance before (2013–2016) and after a large hybridization event which occurred in 2017. We estimated the mean and variance in the landlocked population sizes. We then assessed the directional trend by fitting a simple linear regression and tested for significance. Finally, we also calculated Pearson's correlation coefficient to assess the direction of the relationship between year and landlocked abundance.

### 2.4 Juvenile Growth Rates

We compared growth rates of anadromous juveniles from Rogers Lake to juveniles collected in the progenitor Bride Lake before recruitment to the ocean. After genetic assignments of juveniles to ecotype and hybrid classes, otoliths were also removed and aged for a subset of individuals as described in Littrell et al. (2018). We aged 275 anadromous juveniles from Rogers Lake between 2016 and 2021 by counting daily growth increments in sagittal otoliths, with samples prepared using resin mounting and fine polishing. A Leica DM LS2 compound microscope was used to count daily otolith increments at 20×–40× magnification. In addition, 324 Bride Lake juveniles were aged in 2015 (Littrell et al. 2018) and 2017, which allowed for growth rate comparisons between Bride Lake juveniles and Rogers Lake juveniles. Absolute growth was calculated with L = α + g, where L is the Total Length (mm), α is the age from ring counts derived from otolith daily deposits and g (mm/day) is the mean daily growth. Water temperature was also recorded in both Rogers and Bride Lakes using a YSI ProSolo handheld optical dissolved oxygen meter (YSI Inc.). Measurements were taken in 0.5 m increments and averaged from the upper three meters in the deepest part of each lake.

To assess the growth rates of the landlocked and hybrid juveniles in comparison to the anadromous juveniles in Rogers Lake through the duration of the secondary contact, we aged 201 landlocked juveniles and 197 hybrid juveniles, as described above and derived the growth rates with the same calculations.

### 2.5 Origin of Returning Anadromous Adults

To identify whether the anadromous adults that were returning up the Rogers Lake fishway (Figure 2A) had either naturally colonized from Mill Brook or were offspring from stocked adults of Mill Brook and Bride Lake origin, we conducted parentage analyses and followed the same approach and criteria as the parentage analyses conducted for the juveniles sampled each year. The Rogers Lake fishway and trap were monitored each day from 2019 to 2022 during the spring run; sex and length were recorded, and a fin clip was sampled from each fish before being released into the lake. After the restoration efforts, the first adult fish migrating to Rogers Lake were detected in 2019. In 2022, a fish counter was placed at the fishway to record the fish entering Rogers Lake. Of the fish returning between 2019 and 2022, 1368 had DNA extracted, were genotyped, and filtered by the same criteria as the annually stocked adults and annually sampled juveniles. We assessed the genetic ancestry of each returning adult to identify if they were purely anadromous, as described above for the juveniles. We asked if any individuals in the dataset were likely to be iteroparous by doing a matching genotypes analysis. Finally, we ran the parentage analyses as described above to ask if the individuals returning were recruited from Rogers Lake stocking events between 2015 and 2018.

### 2.6 Age Structure of Run

To identify the age of the returning adults, and in turn, the composition and structure of the restored population, we summarized the parentage assignments and stocking dates. Age was determined by the stocking date of the assigned parents and the return date of their adult offspring. We compared the age structure proportions among return years to evaluate the overall age structure of the restoring run.

### 2.7 Life‐History Patterns

To measure reproductive success of the stocked adults, we genetically identified and enumerated their offspring from among the adult fish that migrated into Rogers Lake in subsequent years. We assessed if the sex of the parent and the stocking date of the parent influenced the age of and day of lake entry of the offspring.

The stocking dates of anadromous adults reflected distinct return phenology groups, as stocking into Rogers Lake occurred on the day adults entered Bride Lake from the ocean. We therefore tested whether offspring return timing differed among parental stocking groups to determine whether adult migration phenology was associated with offspring migration timing. To test for differences in return dates of offspring among the parental stocking groups from 2017 stocked parents, parents were coded as early (E) 03/30/2017, middle (M) 04/12/2017, and late (L) 04/19/2017 based on stocking date. We conducted each analysis separately by parental sex, offspring sex, and offspring age class. As offspring return‐date distributions deviated from normality, we implemented the nonparametric Kruskal–Wallis rank‐sum test. If the test was significant (*p* < 0.05), we conducted pairwise Wilcoxon rank‐sum tests with Benjamini–Hochberg correction to control for multiple testing. We also assessed the magnitude and direction of differences in return timing by calculating the difference in mean offspring return date between the late and early parental stocking groups; positive values indicate that offspring of late‐stocked parents returned later in the spawning run.

Finally, we also used sibling relationships derived from the parent‐offspring assignments to construct networks in R and assess relationships between parent stock dates and juvenile age of return. We generated undirected networks where each node represented an individual offspring, and edges represented sibling relationships; nodes were scaled by the number of connections. Networks were constructed using igraph (Csardi and Nepusz 2006). Each network was visualized with a force‐directed layout (Fruchterman–Reingold algorithm) to place connected juveniles closer together, allowing us to observe family clustering. Network metrics were used to identify the largest family clusters and average full‐ and half‐sibling clusters by stocking date and year.

## 3 Results

### 3.1 Anadromous Juveniles in Rogers Lake

We detected anadromous juveniles in each year from 2016 onwards (Figure S2). The proportion of anadromous juveniles never exceeded 8% of the total juvenile collection and was always the smallest proportion among the identified genotypes (Figure S3). Stocking increased each year until 2020 (Figure 2A), but the proportion of anadromous juveniles captured did not (Figure 2B). We were able to assign parents to anadromous juveniles from 2016 (4), 2017 (88), and 2018 (65). Parents assigned to anadromous juveniles in 2017 and 2018 were mostly from adults stocked in the middle and last stocking dates (2017 = 62.3% and 2018 = 69.6%; Table 3).

**TABLE 3:** Anadromous offspring produced by stocking date in 2017 and 2018.

Year	Stocking dates			
2017		03/30/17	04/12/17	04/19/17
	03/30/17	12.9%	15.3%	9.4%
	04/12/17		32.9%	24.7%
	04/19/17			4.7%
2018		04/03/18	04/12/18	04/20/18
	04/03/18	4.7%	14.2%	11.1%
	04/12/18		28.5%	26.9%
	04/20/18			14.2%

### 3.2 Juvenile Abundance

Our estimates of juvenile abundance in Rogers Lake (all genotypes) ranged from a low of 61,000 (2025) to a high of 441,667 (2017) individuals (Figure 2B; Tables S3 and S4). The variance of abundance was ~6 times higher prior to 2017 than after 2017 (*p* = 0.05), possibly reflecting the portfolio effect of having multiple genotypes contributing to juvenile abundance after 2017.

From 2016 to 2021, our annual estimates of the abundance of juvenile anadromous alewives ranged from ~1500 in 2016 to ~68,500 in 2017. There was considerable interannual variation across years (CV = 1.36), with no evidence for a directional trend in abundance over the study period (Pearson's correlation between year and abundance; *r* = −0.39; *p* = 0.44).

The abundance of landlocked alewives in Rogers Lake fluctuated substantially, ranging from ~53,000 individuals in 2020 to ~431,000 in 2015. The Landlocked form was the most abundant in the earliest stages of the restoration of anadromous access but was replaced by hybrids by 2021. The mean abundance prior to 2017 was substantially higher (218,670) compared to the post‐2017 period (157,308), and the median declined from 175,563 to 96,282 for the same period. The variance in abundance estimates was also greater before 2017 (2.31 × 10^10^). A general downward trajectory in landlocked abundance was observed over the 9‐year interval, with an estimated negative slope of ~23,000 fish per year. Collectively, these results indicate that landlocked abundance exhibited both temporal variability and a long‐term decline, with stronger evidence for decreasing abundance after 2017. Hybrid offspring were first identified in 2017, and their abundance increased thereafter, with estimates ranging from ~9000 (2019) to ~75,000 (2021) individuals annually (Figure 2B).

### 3.3 Juvenile Growth Rates

Juvenile anadromous alewife spawning in Rogers Lake had absolute growth rates ranging from 0.893–1.168 mm/day (Figure 3). That was a much faster growth rate than juvenile anadromous alewife from Bride Lake (0.575–0.637 mm/day), which have the same anadromous ancestry as the Rogers Lake anadromous alewife (Table S3). Bride Lake has greater densities (with abundance estimates ranging from 177,000 juveniles in 2019 to 1.16 million offspring in 2021; Table S3) than Rogers Lake, indicating increased local competition in Bride Lake. Annual broad‐scale temperatures recorded in the two lakes were highly similar (unpublished data).

**FIGURE 3:**
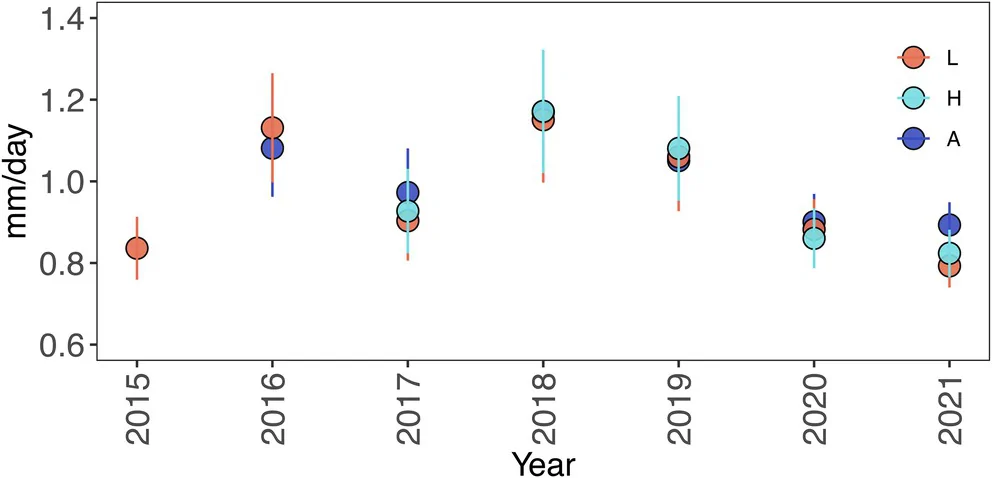
Growth rate comparisons among anadromous (A), hybrids (H), and landlocked (L) juveniles in Rogers Lake during the duration of the study. Growth rates are derived from otolith aging and length.

Landlocked juveniles within Rogers Lake had growth rates that ranged from 0.836 to 1.15 mm/day during the duration of the study. Hybrids produced in the lake had similar growth rates to the landlocked ecotype, ranging from 0.823 to 1.17 mm/day (Figure 3; Table S2).

### 3.4 Origin of Returning Anadromous Adults

Adult anadromous alewife returned to Rogers Lake each year from 2019 onwards (Figure 2A). The period of arrival varied annually, with 2019 being the shortest (02 April to 26 April) and 2020 being the longest (12 March to 21 May; Figure 4). The parentage analysis confirmed that, of the returning adults genotyped between 2019 and 2022, 78% were progeny of adults stocked into Rogers Lake, with a low of 77% in 2022 (10/13) and a high of 97.5% in 2019 (278/285). Of the returning adults identified as produced in Rogers Lake, one was the offspring of adults stocked in 2015, four from adults stocked in 2016, 1115 from adults stocked in 2017, and 80 from adults stocked in 2018 (Figure S4).

**FIGURE 4:**
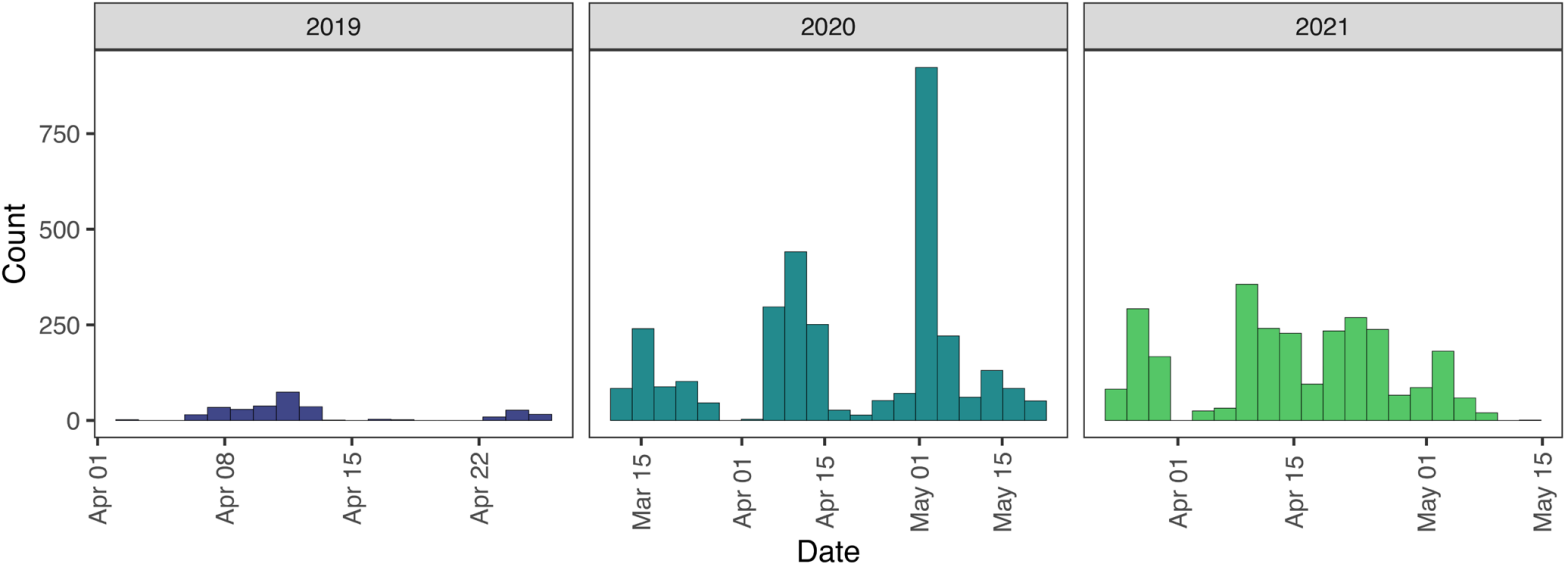
Run duration and count for 2019, 2020, and 2021 of naturally returning fish up the final fishway into Rogers Lake.

The ancestry proportion estimates from STRUCTURE and parentage analyses identified nine returning adults that had hybrid ancestry. All had at least 49% anadromous ancestry and were identified as either anadromous backcross, F1, or F2 hybrids, indicating they had at least one anadromous or F1 parent.

### 3.5 Age Structure of Returning Adults

In each year, the returning adults were dominated by a single age class. In 2019, 99.2% of the returning adults were two years old (276/278) and 0.71% (2/278) were three years old (Figure S4). In 2020, 98% of the returning adults were three (442/450), six were two, one was four, and one was five years old. In 2021, 83% (337/407) of the returning adults were four, 17% were three (69/407), and one was five. Finally, of the 13 individuals genotyped from 2022, seven were five and three were four years old; the remaining fish could not be aged as they had no parents assigned. There was a significant skew in the age and sexes of returning fish, with males comprising 78% of age‐two adults. Most of the returning hybrids with parents that were assigned were identified as three‐year‐old males (~62%), with one male returning as a four‐year‐old. The sexes of the parents producing the returning hybrids were equally male and female. Only two iteroparous individuals were identified and both were males. One returned at two and then again at age three, and the second fish returned at age three and age four (Figure S5).

### 3.6 Life‐History Patterns

Reproductive success differed significantly for adults stocked in 2017 and 2018. In 2017, 34% of the adults produced offspring that returned to Rogers Lake as adults (961/2787 parents producing 1115 returning offspring). In 2018, only 4% of the adults produced offspring that returned to Rogers lake as adults (138/3392 producing 80 returning offspring) (Tables 2 and 4).

**TABLE 4:** Anadromous adult returns produced by stocked parents from 2017.

Return year	Stocking dates			
2019		03/30/17	04/12/17	04/19/17
	03/30/17	46.1%	32.2%	4.76%
	04/12/17		12.8%	3.6%
	04/19/17			0.36%
2020		03/30/17	04/12/17	04/19/17
	03/30/17	24.8%	34.1%	9.0%
	04/12/17		19.9%	9.7%
	04/19/17			2.2%
2021		03/30/17	04/12/17	04/19/17
	03/30/17	16.4%	26.8%	10.1%
	04/12/17		26.5%	16.4%
	04/19/17			3.64%

The stocking date of adults in 2017 had a significant effect on the age of return for their offspring (Figure 5). Nearly all adults returning at age two were from adults stocked earlier in the season in 2017 (Figure 5A). The stocking date of males was significantly associated with the return timing of their male offspring across multiple age classes, with strong effects for ages three and four returns (Table 5). Mean return date was generally later for offspring of later‐stocked fathers, with age‐three and four male offspring of later‐stocked fathers returning an average of 16.9 and 8 days later, respectively, than offspring of early‐stocked fathers (Table 5). Similarly, there was a significant difference in return timing for age‐three and age‐four female offspring, which returned an average of 12.8 and 8.6 days later, respectively, than offspring from early stocked fathers. Pairwise comparisons revealed significant differences between the early (E) and later stocking groups (M and L) but not among later stocking groups (M and L). Associations with maternal stocking dates were weaker and less consistent (Table 5). Overall, the results indicated that later stocking dates were associated with later return timing of offspring (Figure 6), especially due to male stocking dates.

**FIGURE 5:**
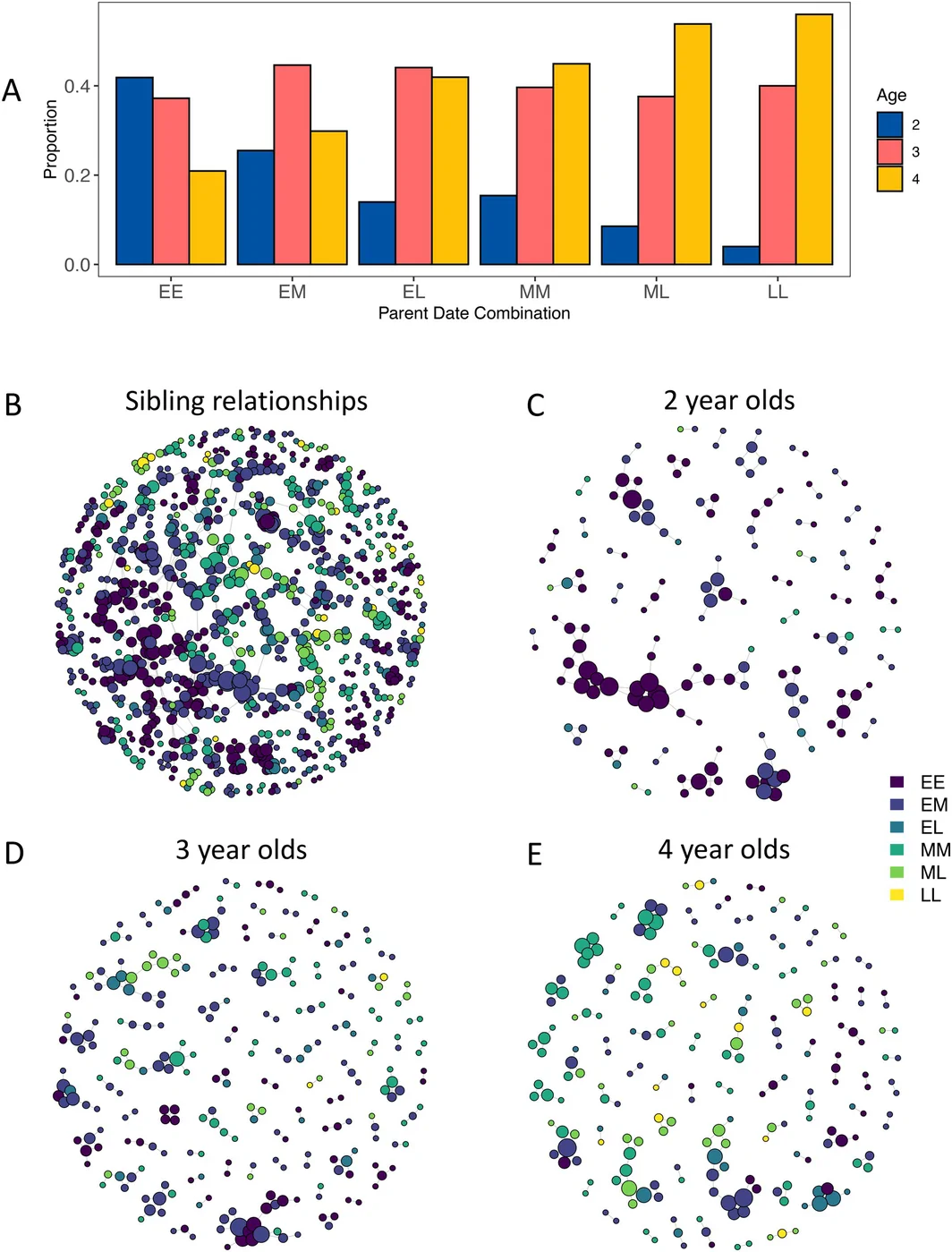
Age and year of return by stocking date for the large 2017 cohort. (A) Proportion of ages by parent stocking combinations of adults and (B) half‐sibling age of return networks by stocking dates. (C) Age two, (D) age three, and (E) age four. Each circle in the network represents a juvenile and the lines connecting two juveniles represent a parent. Circles are color coded by the parent stocking date combination. E is stocking date 03/30/2017, M 04/12/2017 and L is 04/19/2017. Code combinations (EE, EM, EL, MM, ML, and LL) indicate stocking date combinations of both assigned parents.

**TABLE 5:** Association between parental stocking date and offspring return timing.

Parental sex	Offspring sex	Age	*N*	KW *χ* ^2^	*p*	Mean shift (days)
F	F	2	56	0.77	0.679	−1.6
F	F	3	134	5.22	0.074	6.2
F	F	4	154	4.67	0.097	6.7
F	M	2	199	7.4	**0.025**	0.6
F	M	3	290	21.42	**0.000**	14.8
F	M	4	214	0.8	0.669	2.2
M	F	2	57	2.35	0.126	NA
M	F	3	138	16.57	**0.000**	12.8
M	F	4	155	12.65	**0.002**	8.6
M	M	2	203	7.56	**0.023**	1.2
M	M	3	301	32.35	**0.000**	16.9
M	M	4	214	14.74	**0.001**	8

*Note:* *N* indicates the total number of offspring; KW Kruskal–Wallis chi‐squared (*χ* ^2^) statistic; Bold *p*‐values significance indicate (*p* < 0.05) and the mean shift is the mean difference in return date between offspring of early and late stocked parents. NA mean shift could not be calculated due to sample size.

Abbreviations: F, Female; M, Male.

**FIGURE 6:**
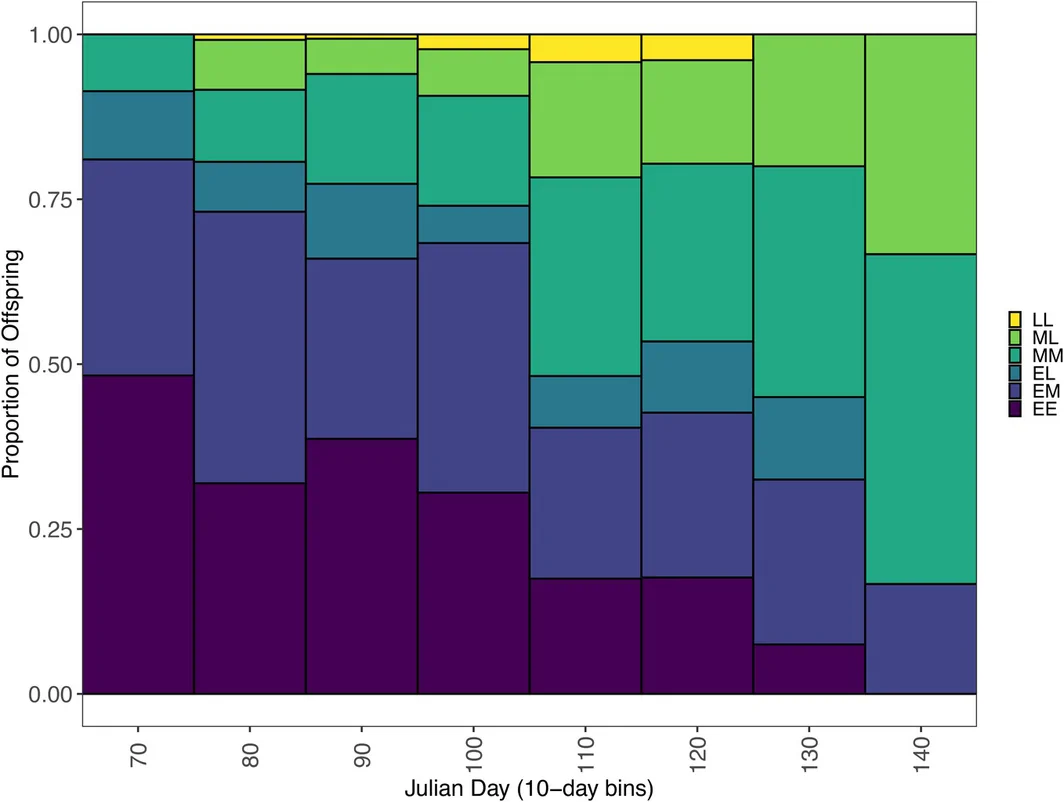
Proportion of individuals returning to Rogers Lake by Julian day interval and the stocking date of the parents. E is stocking date 03/30/2017, M 04/12/2017, and L is 04/19/2017.

In 2017, males from the first stocking date produced more offspring than females stocked on that date (Table 2), because early stocked males participated in additional matings with females from the second stocking date (71% of first to second stocking date matings). Across all 2017 cohorts, we identified a total of 1097 connected relatives, forming a complex, kinship network (Figure 5B). These networks revealed assortative clustering by parental stocking date, particularly in cohorts returning at age three and four (Figure 5C–E). Approximately 35% of half‐siblings were produced from parents stocked on different dates (i.e., 2019, 32%; 2020, 35% and 2021, 38%), indicating repeated breeding within a season of the most successful adults detected (Figure 5B; Tables 4 and 6). Return dates of full‐siblings within the same year were significantly correlated (Figure 7; *r* = 0.41, *p* < 0.001), indicating a likely heritable component to this life‐history trait.

**TABLE 6:** Sibling relationships of returning fish.

Stocking	Year of return	Offspring	Ave full‐sib	Ave half‐sib	Sib group
EE	2019	125	0.328	3.552	
	2020	112	0.366	2.857	
	2021	63	0.253	2.634	
		300			10
EM	2019	87	0.172	3.747	
	2020	154	0.220	3.227	
	2021	102	0.225	3.039	
		343	0.209	3.303	13
EL	2019	13	0.076	3.769	
	2020	40	0.1	2.875	
	2021	39	0.07	2.846	
		92			10
MM	2019	35	0.228	1.714	
	2020	88	0.227	2.136	
	2021	101	0.099	2.435	
		224	0.169	2.205	9
ML	2019	10	0	2	
	2020	44	0.227	2.522	
	2021	59	0.169	1.949	
		113	0.176	2.176	8
LL	2019	1	0	2	
	2020	10	0.1	0.9	
	2021	14	0.071	2.357	
		25	0.08	1.76	6

*Note:* Stocking is the stocking combination of parents, Offspring is the total offspring identified to these stocking combinations by year, Ave full‐sib is the average number of full sibling offspring, Ave half‐sib is the average number of half siblings, and sibling group is the largest sibling group for the combination of parent stocking dates. E is stocking date 03/30/2017, M 04/12/2017, and L is 04/19/2017.

**FIGURE 7:**
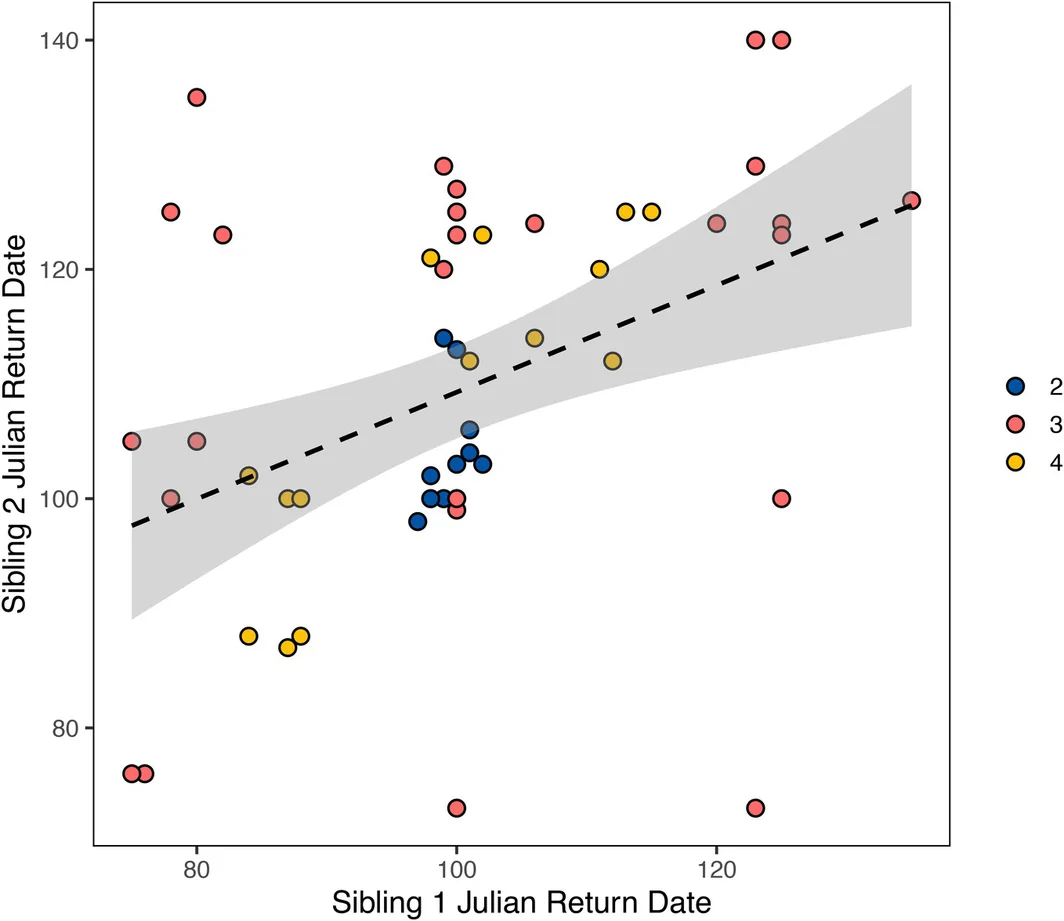
Relationship between full‐sibling pair return dates within years (2019 (2), 2020 (3), and 2021 (4)).

## 4 Discussion

The restoration of anadromous alewives to Rogers Lake highlights both the successes and challenges of reestablishing an anadromous alewife run in a lake that formerly harbored only the landlocked form. The aim of the restoration was to restore the anadromous life‐history to Rogers Lake, with success characterized by annual natural returns occurring independently of continued stocking. Anadromous juveniles were produced in each year in which stocking of anadromous adults occurred. However, the proportion of anadromous juveniles never exceeded 8% of the total juvenile composition for the years 2015–2021, even though the number of anadromous spawning adults increased annually during this period. While the natural adult returns indicate successful reestablishment of the anadromous life‐history, the low proportions of anadromous juveniles produced annually suggest that the long‐term persistence and the increase in size of the run through time remain uncertain. In addition, this may indicate that other factors in the lake, such as density‐dependent mortality or intense competition with the ecologically established landlocked form, are limiting the numbers of anadromous juveniles produced annually (Palkovacs et al. 2008). Anadromous juvenile production may be limited by thermal phenological mismatches (Asch et al. 2019), predation, and constraints imposed by continued inter‐ecotype mating dynamics (Reid et al. 2020).

The divergence of the landlocked form has been driven by unique ecological traits, including their influence on the freshwater zooplankton communities (Post et al. 2008; Palkovacs et al. 2008; Palkovacs and Post 2008, 2009; Palkovacs, Hasselman, et al. 2014). These prior observations indicate that, while the two alewife ecotypes differ in their preferred prey when found in separate lakes, they may compete for resources when interacting within the same lake. Similar limitations have been observed in other fish restoration efforts, where population establishment is then limited by biotic factors and density‐dependence (Jonsson and Jonsson 2009).

We observed a ~50% growth advantage in anadromous juvenile alewives from Rogers Lake, relative to juveniles in the progenitor Bride Lake. This is potentially linked to density dependence in Bride Lake, which previous work has shown restricts juvenile growth due to resource competition (West et al. 2010). The low densities of anadromous juveniles in Rogers Lake may allow anadromous juveniles to achieve growth rates that are similar to the ecologically locally adapted landlocked form and hybrids. In addition, competition with landlocked and hybrid juveniles for shared resources and differences in prey communities among lakes may also influence growth, although observed differences in habitat use among ecotypes in Rogers Lake may limit competition (Jones et al. 2013; Reid et al. 2020; Guo et al. 2021; Devine et al. 2024). Lake temperature has been shown to influence juvenile alewife growth (Guo et al. 2021). However, broad‐scale temperature profiles are highly similar between Rogers and Bride Lakes, making temperature an unlikely cause of growth differences in this case. The increased growth rate observed in Rogers Lake juveniles likely has important implications for the restoration efforts by influencing the age of maturation and portfolio of life‐history strategies in the anadromous run.

The first years of anadromous returns (2019–2022) were dominated by a single cohort from parents stocked in 2017. This cohort was estimated at ~68,500 juveniles and was responsible for 92% of the returns assigned to stocked parents. In addition, the network analyses further point to the skew in reproductive success and that only a small proportion of stocked adults contributed to this large cohort, and in turn, the genetic and phenotypic diversity of the reestablished anadromous run. These results indicate that the conditions for successful recruitment of anadromous alewives are highly variable.

We observed anadromous adults returning to Rogers Lake at slightly younger ages compared to well‐established alewife runs in the region, as well as other reestablished runs in lakes without a landlocked component (Havey 1961). A shift in age of return has been observed in fish returning to Bride Lake, from predominantly five‐ to seven‐year‐olds (1960s), to predominantly three‐ and four‐year‐olds in the early 2000s (Davis and Schultz 2009). The demographic shift to smaller returning adult body size, indicative of declining maturation age, has been observed across the alewife range (Palkovacs, Mandeville, and Post 2014). We also observed much lower rates of iteroparity than estimated in Bride Lake (Davis and Schultz 2009). These patterns suggest that the faster freshwater growth achieved in Rogers Lake allows juvenile alewives to meet the required size threshold for seaward migration more quickly (Gahagan et al. 2010), which in turn may lead to earlier maturation and younger age at return as observed in other anadromous species (Scheuerell 2005). If so, then this effect may accelerate demographic recovery by increasing the population growth rate in the early stages of restoration. In addition, we found that the stocking date of anadromous adults was associated with reproductive success as well as age at maturation and day of return of their progeny, indicating that stocking practices and the migration phenology of stocked adults can influence restoration outcomes. Earlier stocking may enhance success by allowing adults more time to recover from migratory stress and mature gonads under favorable conditions (Marjadi et al. 2019, 2023). It should be noted that migration and spawn timing may be decoupled in some years and vary among populations (Rosset et al. 2017) and therefore factors such as freshwater residence time and mate availability may also contribute to the patterns we observe. In addition, as the stocking date in our restoration experiment reflected the parental migration timing, the observed association with offspring return timing suggests that a stocking strategy that maintains the diversity of the migration run will preserve variation in the restored population.

The restoration of anadromous alewives to Rogers Lake has impacted the established landlocked form in the lake, shaping the evolutionary trajectory of the population. Rapid gene flow and introgression of anadromous ancestry into the landlocked population was observed from 2017 onwards. We also observed a general downward demographic trend of the landlocked fish (and total estimated annual juvenile abundance) since 2017, suggesting that the restoration of the anadromous component is impacting the demographic trajectory of the landlocked form. This decline could be an indication of competition, as the landlocked form now shares resources with the anadromous juveniles and hybrids. While prior work shows that the two ecotypes differ in their preferred prey resources when in isolation, their diets do overlap (Palkovacs and Post 2008; Jones et al. 2013) suggesting the potential for competition when they occur in the same lake.

Although landlocked abundance declined following the restoration of anadromy, hybrid abundance in the lake appears to be increasing, as hybrid juveniles were identified in increasing numbers every year since 2017, although F1 hybrids were rare after 2017. This indicates that a single year of extensive hybridization between anadromous and landlocked adults initiated rapid directional introgression into the landlocked population. Despite the demographic shifts within the lake, growth rates of the landlocked and hybrid juveniles remained consistent before and after the reestablishment of anadromous fish. Hybrid individuals also successfully completed the entire marine life cycle, recruiting to the ocean and returning as mature anadromous adults. This pattern reflects the behaviors observed in *Oncorhynchus mykiss* , where resident and anadromous ecotypes frequently interbreed and therefore maintain a polymorphic population (Pearse et al. 2019). The migratory outcome for hybrid alewives may depend on a conditional life‐history strategy, where both the individual condition and the local environmental factors may influence whether the individual migrates or not. The simultaneous decline in landlocked abundance and increase in hybrids suggests that restoration is driving changes in the long‐term evolutionary trajectory of the alewife population and recruitment dynamics in Rogers Lake. This raises important questions about the long‐term evolutionary trajectory of the system. Hybridization may either change the genetic and phenotypic distinctiveness of the landlocked ecotype or may facilitate the maintenance of mixed ancestry and traits from both forms, resulting in a stable hybrid component to the population. Continued monitoring of the genetic and phenotypic composition of Rogers Lake will be necessary to understand the long‐term fate of the landlocked population during the anadromous alewife restoration.

Whole‐lake restoration efforts, such as the reestablishment of anadromous alewives to Rogers Lake, are powerful eco‐evolutionary experiments that provide insights into the dynamics of secondary contact. In systems where migratory and resident forms come into secondary contact, restoration may lead to hybridization, change in life‐history patterns, and altered demographic trajectories. The rapid reestablishment of the anadromous alewife run in Rogers Lake shows that anadromous alewife restoration is possible in lakes that contain landlocked populations, a common scenario in many coastal lakes that are candidates for restoration. Nonetheless, the eco‐evolutionary outcomes of restoration are complex and may ultimately cause the loss of landlocked adaptations that have emerged over the past several hundred years, albeit in response to the construction of the dams that are now candidates for removal or fishway installation (Palkovacs et al. 2008).

Rogers Lake provides an empirical example of these dynamics, highlighting how the success of the restoration and the impacts of stocking practices to restore the run occurred alongside the unexpected outcome of hybridization and rapid introgression. Future restoration projects should account for potential eco‐evolutionary dynamics, integrating demographic monitoring as well as genetic and phenotypic analyses to capture the full range of possible outcomes. These actions will lead to improved restoration strategies and clarify our understanding of how evolutionary processes are influenced by the impacts of human modifications to ecological systems.

## Funding

This work was funded by the National Science Foundation (DEB‐1556378, DEB‐2102763), the Wildlife Research Institute Northeast Regional Conservation Needs Grants Program, the NOAA Cooperative Institute for Marine, Earth, and Atmospheric Systems (CIMEAS), and the Connecticut Department of Energy and Environmental Protection.

## Conflicts of Interest

The authors declare no conflicts of interest.

## Supporting information


**Figure S1:** Mill Brook and Bride Brook run counts for 2004 to 2018, showing how low the run counts for Mill Brook were at the start of the restoration in 2015 (134 fish) and onwards in comparison to Bride Brook, one of the strongest runs in the region.
**Figure S2:** Structure plots 2015–2021 showing proportion of ancestry, dark blue anadromous (ANAD) and light blue landlocked (LAND).
**Figure S3:** Proportion of genotyped individuals designated to landlocked (Land—orange), hybrids (Hybs—yellow) and anadromous (Anad—blue). By 2021 the proportion of individuals caught were mostly designated as hybrids.
**Figure S4:** The distribution of the returning anadromous run which were genetically aged. Samples were selected to represent the entire run of each year.
**Figure S5:** Distribution of related individuals and iteroparous individuals in the dataset. Only two individuals were identified as iteroparous.
**Table S1:** Adult alewife entering Rogers Lake through the Rogers Lake Fishway. From 2022 these estimates are based on an installed fish counter.
**Table S2:** Abundance estimates broken down by ecotype and hybrids (“intermediates”) as well as total abundance estimates with upper and lower confidence estimates.
**Table S3:** Juvenile metadata based on density estimates, length, and otolith ages.

## Data Availability

The data that support the findings of this study will be openly available with Dryad at DOI: 10.5061/dryad.59zw3r2pm.
