# Supplementary material for: Eco‐Evolutionary Dynamics of Anadromous Alewife Restoration in a Whole‐Ecosystem Experiment
Source: Evol Appl. 2026 Nov 6;19(11):e70350. doi: 10.1111/eva.70350 (PMC13743864; doi:10.1111/eva.70350)
Supplement: Supplementary file 1 [file EVA-19-e70350-s001.pdf]

# Supplementary Material

## Eco-evolutionary dynamics of anadromous alewife restoration in a whole-ecosystem experiment

**Supplementary Figures**

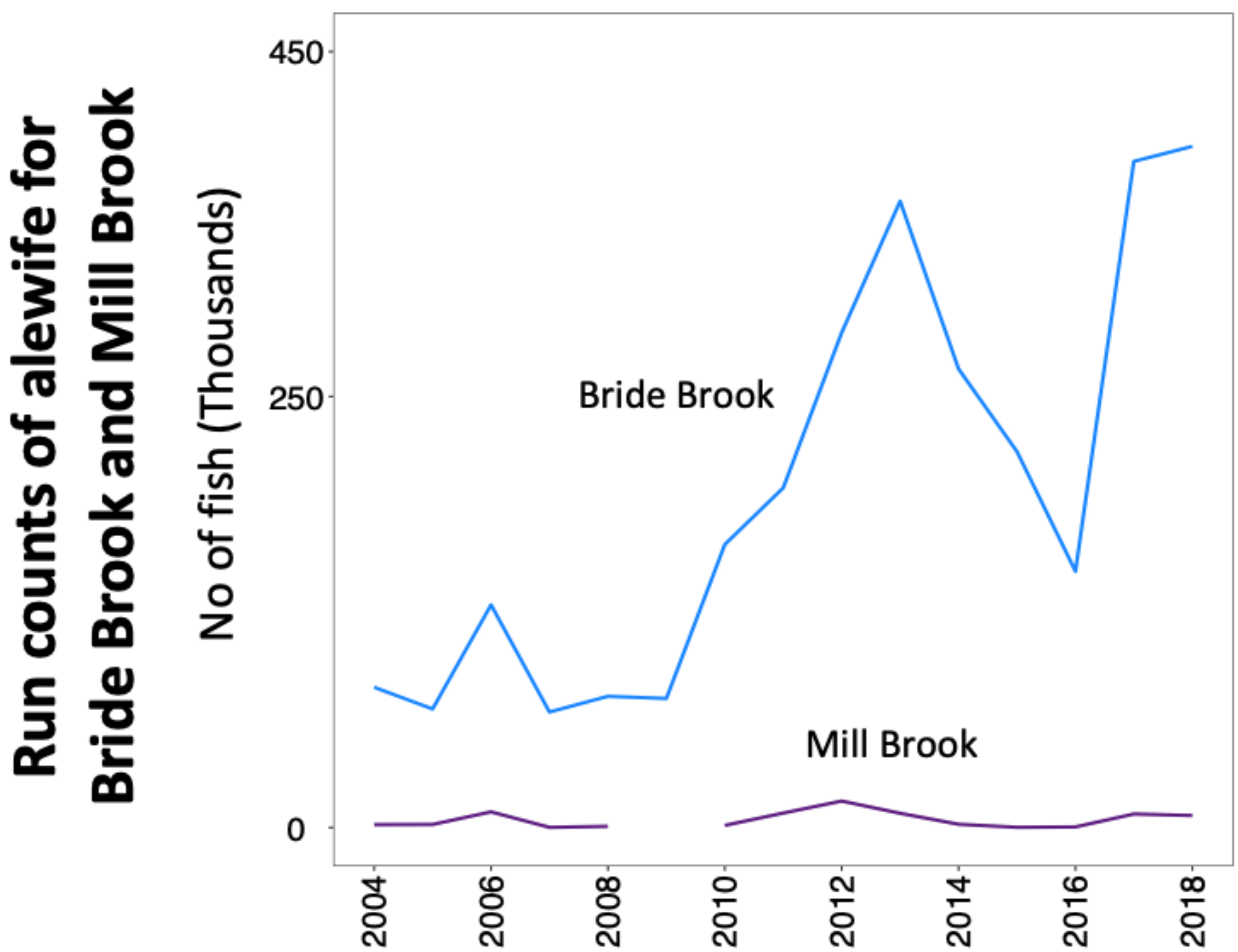


Fig. S1 Mill Brook and Bride Brook run counts for 2004 to 2018, showing how low the run counts for Mill Brook were at the start of the restoration in 2015 (134 fish) and onwards in comparison to Bride Brook, one of the strongest runs in the region

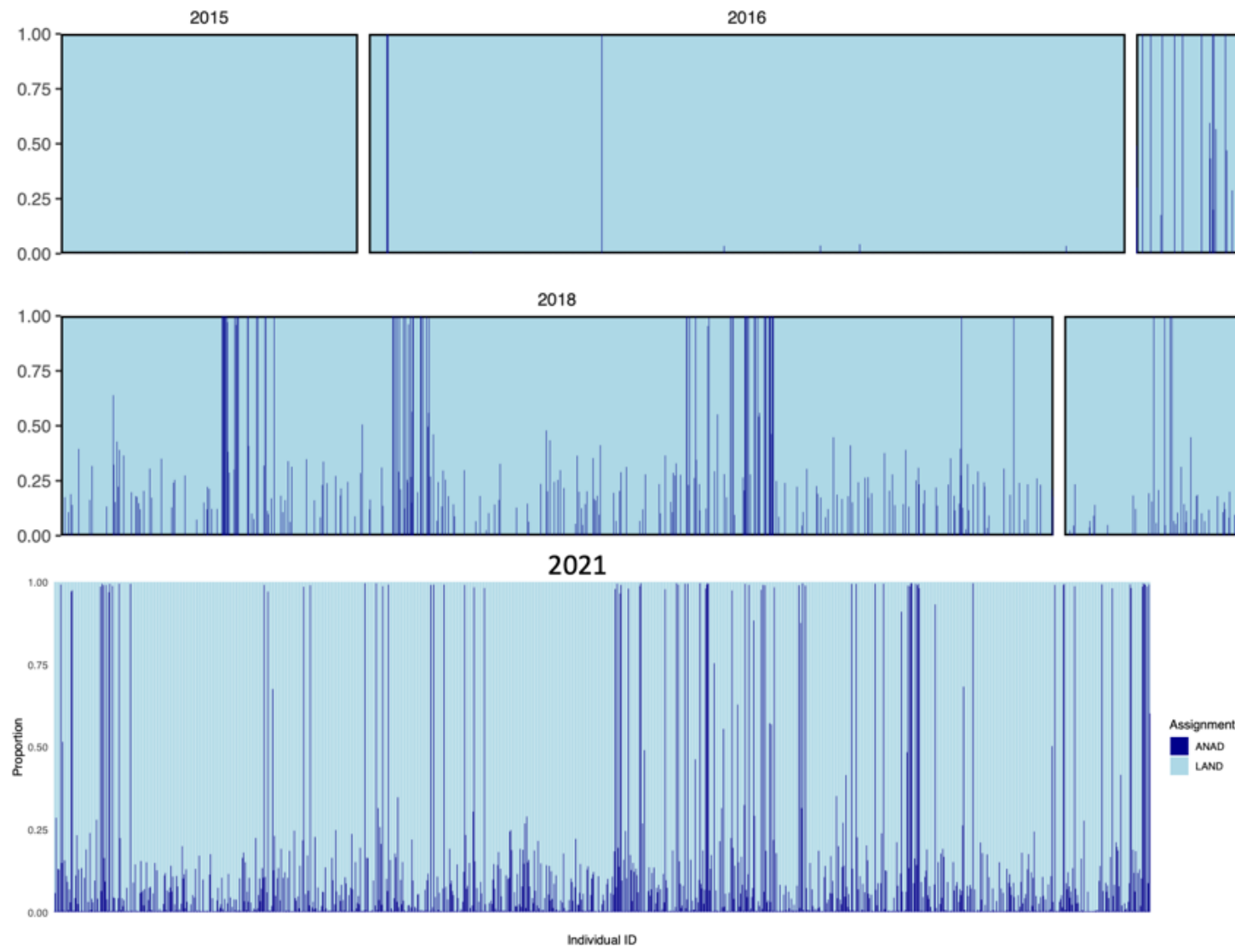


Fig. S2 Structure plots 2015 to 2021 showing proportion of ancestry, dark blue anadromous (ANAD) and light blue landlocked (LAND)

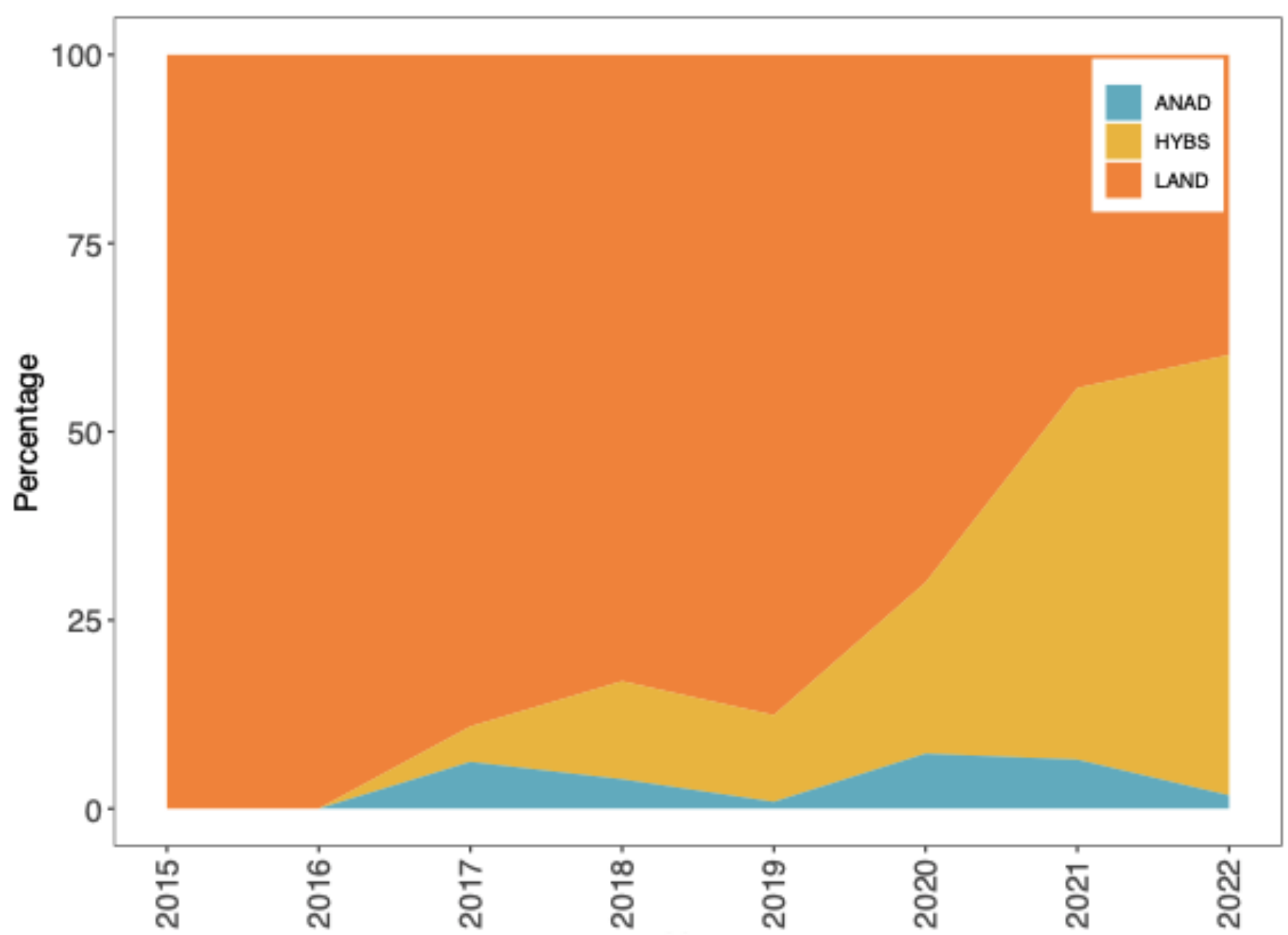


Fig S3 Proportion of genotyped individuals designated to landlocked (Land – orange), hybrids (Hybs – yellow) and anadromous (Anad – blue). By 2021 the proportion of individuals caught were mostly designated as hybrids

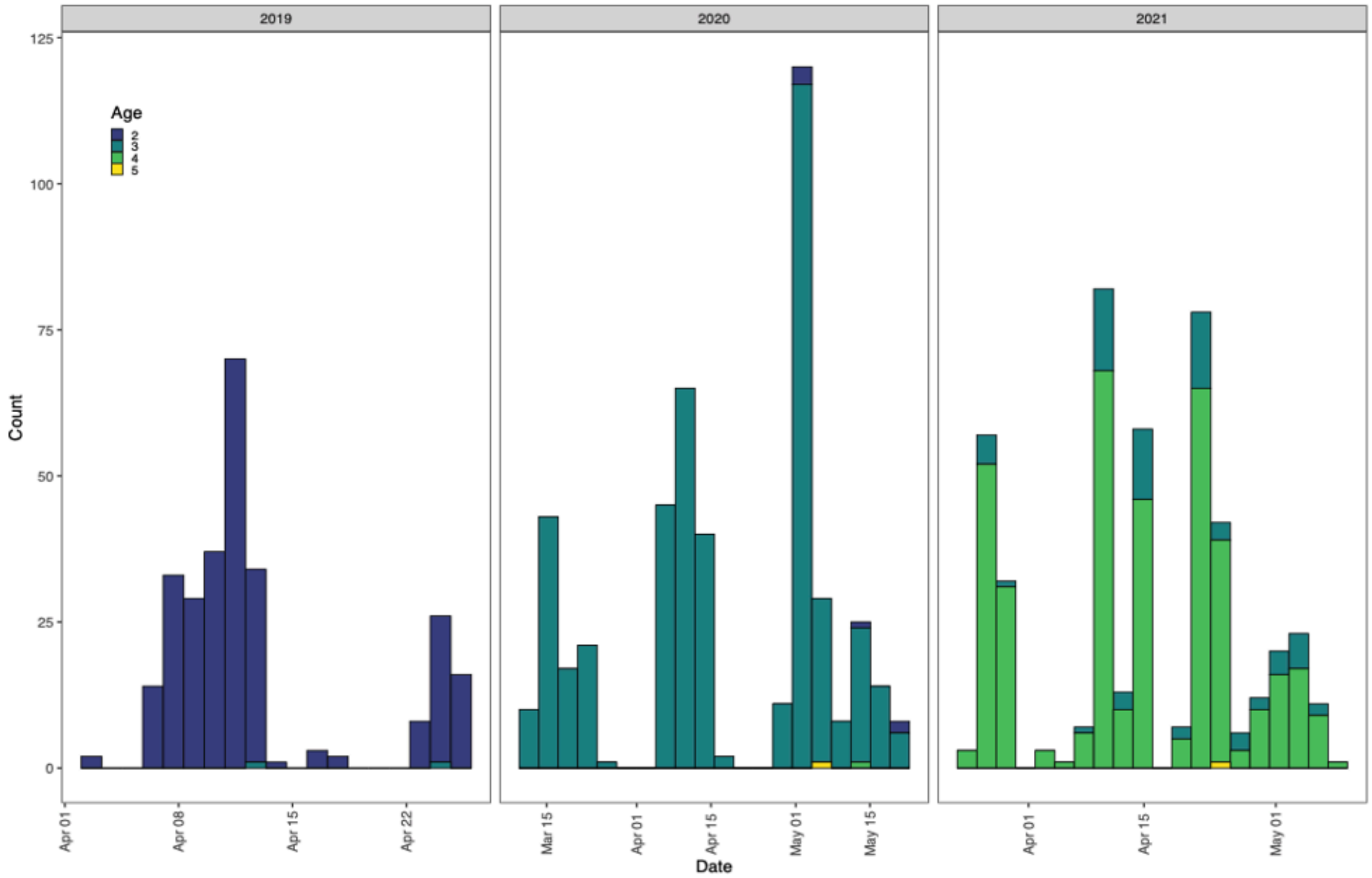


Fig S4 The distribution of the returning anadromous run which were genetically aged. Samples were selected to represent the entire run of each year.

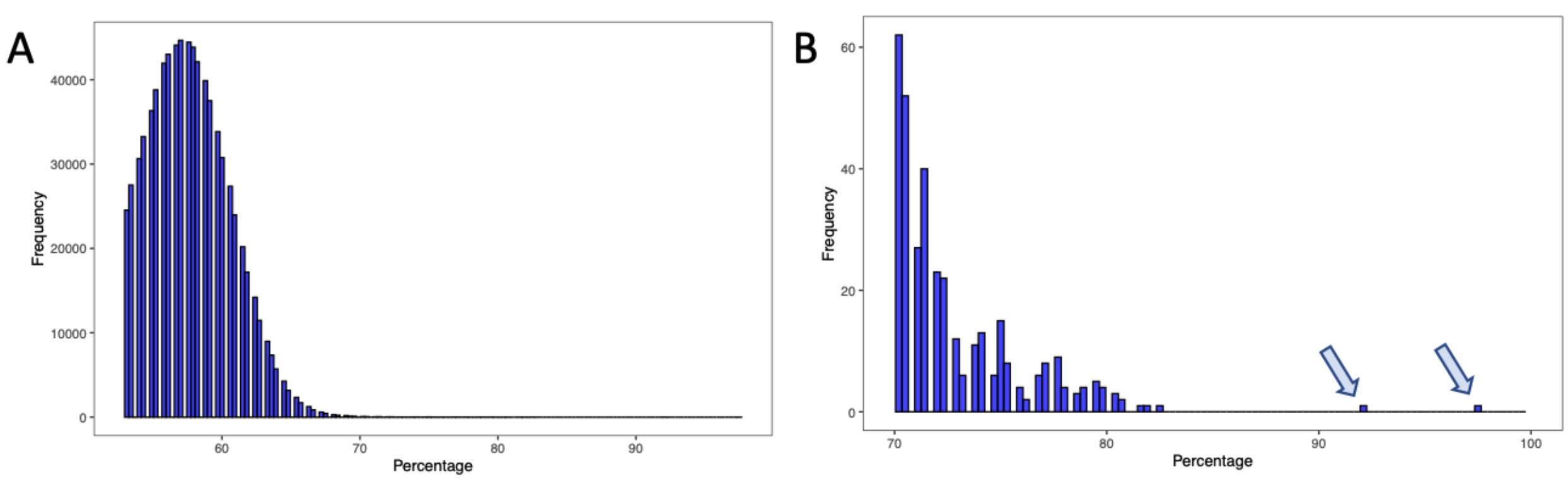


Fig. S5 Distribution of related individuals and iteroparous individuals in the dataset. Only two individuals were identified as iteroparous.

# Supplementary Tables

Table S1 Adult alewife entering Rogers Lake through the Rogers Lake Fishway. From 2022 these estimates are based on an installed fish counter

| Year | Total Returns |
|---|---|
| 2019 | 286 |
| 2020 | 2842 |
| 2021 | 2506 |
| 2022 | 239 |
| 2023 | 1245 |
| 2024 | 13545 |
| 2025 | 6404 |

Table S2 Abundance estimates broken down by ecotype and hybrids (“intermediates”) as well as total abundance estimates with upper and lower confidence estimates

| Year | Landlocked | Anadromous | Intermediate | Total | Lower 95% | Upper 95% |
|---|---|---|---|---|---|---|
| 2013 | 223483 | 0 | 0 | 223483 | 122412 | 324555 |
| 2014 | 127642 | 0 | 0 | 127642 | 68382 | 186901 |
| 2015 | 431067 | 0 | 0 | 431067 | 343975 | 518159 |
| 2016 | 92486 | 6448 | 0 | 98933 | 44054 | 153813 |
| 2017 | 350186 | 68527 | 22953 | 441667 | 230013 | 653320 |
| 2018 | 220682 | 15391 | 35817 | 271890 | 212583 | 331197 |
| 2019 | 96282 | 1527 | 9074 | 106883 | 37475 | 176292 |
| 2020 | 53167 | 6244 | 18323 | 77733 | 17638 | 137828 |
| 2021 | 66224 | 9409 | 75417 | 151050 | 57322 | 244778 |
| 2022 | | | | 108650 | 64972 | 152328 |
| 2023 | | | | 190800 | 97519 | 284081 |
| 2024 | | | | 183733 | 81723 | 285743 |
| 2025 | | | | 60950 | 30754 | 91146 |

Table S3 Juvenile metadata based on density estimates, length and otolith ages

| YEAR | LAKE | ECOTYPE | DENSITY(M2) | Pooled_SE | POP_SIZE | AVESIZE_ANAD | AVESIZE_ANAD_SE | AVESIZE_LAND | AVESIZE_LAND_SE | Growth_ANAD_SD | Growth_ANAD_SD | Growth_LAND_SD | Growth_LAND_SD |
|---|---|---|---|---|---|---|---|---|---|---|---|---|---|
| 2015 | BRIDE | ANAD | 1.16 | 1.20 | **336081** | 42.497 | 5.989 | | | 0.575 | 0.077 | | |
| 2016 | BRIDE | ANAD | 1.01 | 0.66 | **292803** | 43.970 | 6.651 | | | | | | |
| 2017 | BRIDE | ANAD | 0.36 | 0.14 | **104018** | 57.290 | 5.200 | | | 0.637 | 0.077 | | |
| 2018 | BRIDE | ANAD | 1.96 | 1.98 | **569002** | 39.310 | 5.395 | | | | | | |
| 2019 | BRIDE | ANAD | 0.61 | 0.14 | **177093** | 55.400 | 3.533 | | | | | | |
| 2020 | BRIDE | ANAD | 2.33 | 1.21 | **675072** | 40.377 | 4.037 | | | | | | |
| 2021 | BRIDE | ANAD | 4.03 | 0.87 | **1168594** | 50.989 | 5.109 | | | | | | |
| 2022 | BRIDE | ANAD | 1.31 | 0.93 | **378875** | 45.621 | 5.886 | | | | | | |
| 2023 | BRIDE | ANAD | 1.33 | 0.91 | **386261** | 55.818 | 7.569 | | | | | | |
| 2024 | BRIDE | ANAD | 3.22 | 1.06 | **934602** | 40.640 | 6.607 | | | | | | |
| 2015 | ROGERS | LAND | 0.41 | 0.22 | **431067** | | | 27.305 | 8.218 | | | 0.836 | 0.077 |
| 2016 | ROGERS | MIXED | 0.09 | 0.04 | **98933** | 95.000 | 2.160 | 37.690 | 9.827 | 1.081 | 0.119 | 1.131 | 0.134 |
| 2017 | ROGERS | MIXED | 0.42 | 0.17 | **441667** | 76.000 | 4.011 | 66.228 | 5.733 | 0.973 | 0.108 | 0.903 | 0.097 |
| 2018 | ROGERS | MIXED | 0.26 | 0.07 | **271890** | 107.234 | 5.039 | 44.871 | 16.289 | 1.168 | 0.144 | 1.151 | 0.154 |
| 2019 | ROGERS | MIXED | 0.10 | 0.07 | **106883** | 100.714 | 3.546 | 63.810 | 14.092 | 1.051 | 0.109 | 1.060 | 0.133 |
| 2020 | ROGERS | MIXED | 0.07 | 0.07 | **77733** | 86.353 | 12.510 | 64.339 | 12.647 | | | | |
| 2021 | ROGERS | MIXED | 0.14 | 0.09 | **151050** | 82.622 | 7.239 | 65.020 | 8.245 | | | | |
| 2022 | ROGERS | MIXED | 0.10 | 0.05 | **108650** | 100.750 | 3.327 | 36.125 | 10.020 | | | | |
| 2023 | ROGERS | MIXED | 0.21 | 0.09 | **219067** | | | | | | | | |
| 2024 | ROGERS | MIXED | 0.15 | 0.10 | **157233** | | | | | | | | |
